# Clinical Acceptance of Digitally Produced Zirconia and Metal Post and Cores, Based on the Impression Method

**DOI:** 10.3390/clinpract14060199

**Published:** 2024-11-20

**Authors:** Paula Perlea, Cosmin Stefanescu, Alexandru Eugen Petre

**Affiliations:** 1 Department of Endodontics, Carol Davila University of Medicine and Pharmacy, 101221 Bucharest, Romania; paula.perlea@umfcd.ro; 2 Department of Prosthodontics, Carol Davila University of Medicine and Pharmacy, 032799 Bucharest, Romania; alexandru.petre@umfcd.ro

**Keywords:** dentistry, digital, cad-cam, post and core

## Abstract

Background: The existing literature predominantly examines post and core assessments post-cementation, neglecting the critical pre-cementation phase. Research on the clinical acceptance of dental posts received from dental laboratories before cementation is notably lacking. This study investigates the percentage of zirconia and metal dental posts that are deemed suitable for cementation by clinicians, among the total received from the dental laboratory. Additionally, it aims to examine whether this percentage varies based on the type of impression made by the clinician: digital impression versus conventional impression. Methods: This article introduces the application of computer-aided design-computer-aided manufacturing (CAD-CAM) technology for manufacturing customized zirconia and Cobalt–Chromium (Co-Cr) post and cores. Intraoral scanning is employed to capture the canal anatomy. In contrast to the traditional casting process, a three-dimensional (3D) metal printer machine is utilized to 3D print the metal post and core from Co-Cr, resulting in enhanced toughness and superior adaptability to the canal. Two null hypotheses were formulated, investigating the clinical acceptance of zirconia and metal posts obtained through traditional versus digital impressions. Results: Among 577 post and cores, 95% of metal posts from both impression methods received clinical approval. However, for zirconia posts, a significantly higher acceptance rate (95% versus 88%) was observed for those from traditional impressions. The Chi-squared test yielded a *p*-value < 0.05, underscoring the clinical superiority of conventionally obtained zirconia posts and supporting the null hypothesis for metal posts. Conclusions: A significantly higher acceptance rate is apparent among zirconia post and cores manufactured through conventional impressions, in contrast to zirconia post and cores produced via digital impressions. No statistically significant difference was identified between metal post and cores obtained through digital impressions and those acquired through conventional impressions.

## 1. Introduction

Caries and traumatic injuries frequently lead to the significant loss of coronal tooth structure. In instances where the extent of loss is substantial, the inherent natural tooth structure becomes insufficient to adequately support a restoration. Therefore, the treatment with a post becomes mandatory. The preservation of the remaining tooth structure is the most important factor for the long-term success of a tooth subjected to endodontic treatment, regardless of the type [1] or length of the post used [2]. Many esthetic post systems available in the market are dependent on the incorporation of a prefabricated post component into the definitive post. The utilization of a prefabricated post, composed of either composite resin or ceramic materials, necessitates canal preparation to accommodate the size and shape of the post. Subsequently, this is followed by the addition of a composite resin core to the post after cementation, or alternatively, the heat pressing of a ceramic core onto the ceramic post during its fabrication. In both scenarios, the fit of the post within the canal walls relies on the configuration and size of the root canal [3]. In round canals with sufficient thickness of dentinal walls, a prefabricated post can achieve close adaptation to the prepared walls along its entire length. Nevertheless, in wide, noncircular, or highly tapered canals, systems utilizing a cylindrical prefabricated post may struggle to achieve optimal adaptation to the canal, potentially compromising post retention. This article outlines the utilization of computer-aided design-computer-aided manufacturing (CAD-CAM) technology for the yttrium-tetragonal zirconium polycrystals (Y-TZP) system (Luxen, Dentalmax, Cheonan-si, South Korea) in the fabrication of a customized zirconia post and core. The methodology follows similar initial procedures employed for creating a metal post and core, involving the generation of an intra oral scanner pattern to capture the canal’s anatomy. However, instead of the traditional process of investing and casting the post in metal, the metal post and core are 3D printed from Cobalt–Chromium (Co-Cr) using a 3D metal printing machine (MySint100, Sisma, Vicenza, Italy). These two techniques result in a post and core characterized by enhanced toughness and maximum adaptability to the canal.

For numerous years, the favored treatment method consisted of a cast post-and-core. This substructural approach aimed to enhance the strength of the teeth while simultaneously ensuring the necessary retention and resistance for the covering restoration [3].

Different metal alloys, particularly Co-Cr, have been utilized extensively in the manufacturing of customized post and cores [4]. CAD-CAM techniques encompass both subtractive and additive manufacturing methods for custom post and core fabrication [5]. Subtractive methods, involving milling tools, result in material waste and demand precision. The challenges associated with tooling machine access to intricate dental anatomies have led researchers to explore additive manufacturing methods [6]. Various additive manufacturing techniques are employed in the production of metal objects, with selective laser melting (SLM) being one of the most widely utilized technologies [7].

The utilization of CAD-CAM has experienced a notable rise in the construction of indirect restorations. CAD-CAM milling systems have streamlined laboratory procedures, addressing the limitations associated with traditional casting methods. The digital design of post-and-cores through CAD technology enables comprehensive planning of clinical treatments, while the CAM process expedites and precisely fabricates the post-and-core, leading to a substantial reduction in treatment costs [8]. CAD-CAM systems have been utilized for the precision milling of zirconia, facilitating the development of a customized one-piece post-and-core structure designed to fit into elliptical post spaces [9]. Zirconia posts were initially introduced in 1995 as a substitute for cast metal post-and-cores, offering an advantageous option for teeth with significant loss of coronal structures. Considering their elevated translucency and the capacity to harmonize with tooth color, these posts demonstrated superior esthetics, yielding restorations that closely resembled natural teeth [10].

Over a period of 10 years, Calabro et al. [11] demonstrated a success rate of 97.1% for metal posts, concluding that the studied posts exhibited excellent clinical performance. In comparison, over the same 10-year period, Bateli et al. [12] studied the survival of zirconia posts-and-cores and indicated different outcomes, revealing a survival rate of 81.3%.

The fatigue resistance of posts has also been studied. In their study, Altitinchi et al. [13] illustrated comparable fatigue resistance between the cast metal and zirconia post-and-core groups (*p* > 0.05), with both demonstrating significantly higher fatigue resistance than the fiber-reinforced groups (*p* < 0.05). Regarding fatigue resistance, Aktemur et al. [14] investigated cast versus zirconia post-and-cores, finding that the post type significantly influenced both the fracture resistance and retention of the utilized posts. In a meta-analysis involving 844 endodontically treated teeth, Martins et al. revealed no statistically significant differences in terms of root fracture (*p* = 0.44) and debonding (*p* = 0.56) between metal posts and fiber posts [15].

There is a research gap as all existing articles have focused on assessing post and cores after cementation, while none have addressed pre-cementation aspects, specifically, how many of the posts received from the laboratory are accepted for cementation by clinicians and how many are not. This crucial consideration, referred to as clinical acceptance, signifies the percentage of post and cores deemed acceptable by clinicians before cementation.

Moreover, considerable attention has been given to the assistance provided by CAD-CAM technology in dentistry overall, including its application in post and core restorations. Lin et al. [16] described a digital workflow used for metal post-and-core fabrication, integrating digital impressions with an intraoral scanner (IOS) and CAD-CAM. This streamlined workflow enabled the completion of the restoration in a significantly shorter timeframe compared to conventional techniques, promoting more efficient and predictable treatment outcomes while reducing both time and costs for the patient.

This study aims to examine the ratio of zirconia and metal post and cores accepted for cementation by clinicians, out of the total number of post and cores received from the dental laboratory. Moreover, it seeks to investigate if this proportion differs depending on the clinician’s impression type: digital or conventional. This article presents the use of CAD-CAM technology for producing custom zirconia and Co-Cr post and cores.

Therefore, considering the described aspects, a null hypothesis has been formulated: there is no difference in the clinical acceptance of zirconia posts obtained through traditional versus digital impressions. Additionally, another null hypothesis has been postulated: there is no difference in the clinical acceptance of metallic posts obtained through traditional versus digital impressions.

## 2. Materials and Methods

This article details the application of CAD-CAM technology for the Y-TZP system, specifically for the production of zirconia post and cores. The process involves digital initial steps resembling the fabrication of a metal cast post and core, including the generation of an intraoral scan to capture the canal’s anatomy. In contrast to the conventional investment and casting processes for metal, this approach incorporates scanning, milling, and sintering of the Y-TZP. Remarkable advantages include heightened toughness, optimal adaptability to the canal, and favorable esthetic characteristics. The study was conducted in a dental laboratory. The data received from the dentists was analyzed and statistical analysis was performed. There were five dentists who collaborated with the dental laboratory and all of them were specialists in Prosthodontics. In the esthetic zone, clinicians choose zirconia posts and cores to prevent the metal tint from being visible under ceramic crowns. In comparison, they are not preferred for the posterior region, where significant pressure is applied, and metal post and cores are more suitable.

The laboratory manufactured posts for patients who underwent digital impression using intraoral scanner.

1. Following the completion of endodontic treatment, gutta-percha removal from the tooth is performed using burs (Gates Glidden, Pulpdent Corp., Watertown, MA, USA) to achieve the desired post length, ensuring the preservation of the apical seal.
2. The coronal aspect of the tooth is then prepared to eliminate acute angles between the post surfaces and the apical surface of the core. This adjustment facilitates optimal reading by the intraoral scanner at the core-post junction. Special attention is given to ensuring that the core-post junction possesses sufficient width to prevent fracture during the milling of zirconia. Additionally, the post is shaped with rounded internal line angles where it interfaces with the tooth surfaces.
3. A fully digital workflow was implemented for each zirconia or metal post. The digital post impression was captured using an intraoral scanner (TRIOS 4, 3Shape, Copenhagen, Denmark) and the completeness of the digital impression of the prepared root canal was verified on the scanner’s display. Also, the rules of taking a good TRIOS scan were respected according with the manufacturer’s recommendations, and the clinician dried the teeth well before scanning [17].
4. The STL dataset was utilized to design the post and core in CAD software (Exocad 3.1 Rijeka, EXOCAD GmbH, Darmstadt, Germany). The cement gap parameter was set to 50 µm.
5a. After completing the design, the STL files of the post and cores were transmitted to the 5-axis milling machine (CORiTEC^®^ 250i Loader PRO, Imes Icore GmbH, Eiterfeld, Germany) to mill the zirconia disk (Luxen, Dentalmax, Cheonan-si, Republic of Korea). Subsequently, the attachment points of the post to the CAD/CAM disk were cut and smoothed. The posts underwent sintering in a zirconia furnace (AUSTROMAT Series 6, DEKEMA Dental-Keramiköfen GmbH, Salzburg, Germany) for 11 h at a maximum temperature of 1530 °C.
5b. Upon finalizing the design, STL files of the post and cores were transferred to the 3D metal printer (MySint100, Sisma, Vicenza, Italy), employing Cr-Co powder for the laser sintering of metal posts. Subsequently, attachment points of the printed post underwent cutting and smoothing.

The same laboratory manufactured posts for patients who underwent conventional impression recording using silicone.

1. Following endodontic therapy, gutta-percha removal utilized burs (Gates Glidden, Pulpdent Corp., Watertown, MA, USA) to achieve the desired post length, preserving the apical seal.
2. The coronal aspect underwent preparation to eliminate acute angles between post surfaces and the core’s apical surface. This adjustment optimized the flow of impression material, with a focus on ensuring a sufficiently wide core-post junction to prevent fractures during zirconia milling.
3. Impression recording for post and core preparation involved addition silicone impression material (Elite HD, Zhermack, Badia Polesine, Italy). The silicone was injected into the canal with a disposable dispenser. In addition, alginate (Hydrogum 5, Zhermack, Badia Polesine, Italy) recorded antagonists, while bite registration silicone (Occlufast, Zhermack, Badia Polesine, Italy) recorded intermaxillary relations. Arch impressions were promptly sent to the dental laboratory and poured into plaster models (Elite Rock, Zhermack, Badia Polesine, Italy). For assuring the quality and precision of the plaster models, technicians respected the instructions for use from the manufacturer: water/powder ratio of 20 mL/100 g, 12 min working time, 14 min setting time for a 2 h setting expansion of 0.08%.
4. From this point, the workflow became digital, scanning the plaster models with a laboratory scanner (Medit T510, Medit, Seoul, Republic of Korea).
5. The STL dataset facilitated post and core design in CAD software (Exocad Rijeka, EXOCAD GmbH, Darmstadt, Germany). The cement gap parameter was set at 50 µm.
6a. After completing the design, the STL files of the zirconia post and cores were transmitted to the 5-axis milling machine (CORiTEC^®^ 250i Loader PRO, Imes Icore GmbH, Eiterfeld, Germany) to mill the zirconia disk (Luxen, Dentalmax, Republic of Korea). Subsequently, the attachment points of the post to the CAD/CAM disk were cut and smoothed. The posts underwent sintering in a high-speed furnace (AUSTROMAT Series 6, DEKEMA Dental-Keramiköfen GmbH, Salzburg, Germany) for 11 h at a maximum temperature of 1530 degrees.
6b. Upon finalizing the design, STL files of the post and cores were transferred to the 3D metal printer (MySint100, Sisma, Vicenza, Italy), employing Cr-Co powder for the laser sintering of metal posts. Subsequently, attachment points of the printed post underwent cutting and smoothing.

Following the laboratory procedures, the posts were sent to the clinician, who intraorally evaluated each post and core on every patient. If a post did not fit the prepared tooth cavity, no adjustments were made by the clinician to ensure a proper fit. Consequently, a response was provided to the laboratory for each post: accepted or not accepted. Posts that did not fit during the evaluation received a status of “not accepted”. All these responses were gathered into a table, forming a study on post and core acceptability throughout the calendar year: January to December 2023. A total of 577 post and cores were screened for this research and categorized into four groups: Co-Cr metal posts obtained through conventional impressions (Group 1), Co-Cr metal posts obtained through digital impressions (Group 2), zirconia posts obtained through conventional impressions (Group 3), zirconia posts obtained through digital impressions (Group 4). Differences in the proportions of accepted and non-accepted zirconia post and cores, as well as accepted and non-accepted metal post and cores, were assessed using the Chi-square test performed by dedicated statistical software (Prism, GraphPad 9, San Diego, CA, USA).

## 3. Results

**Figure 1:**
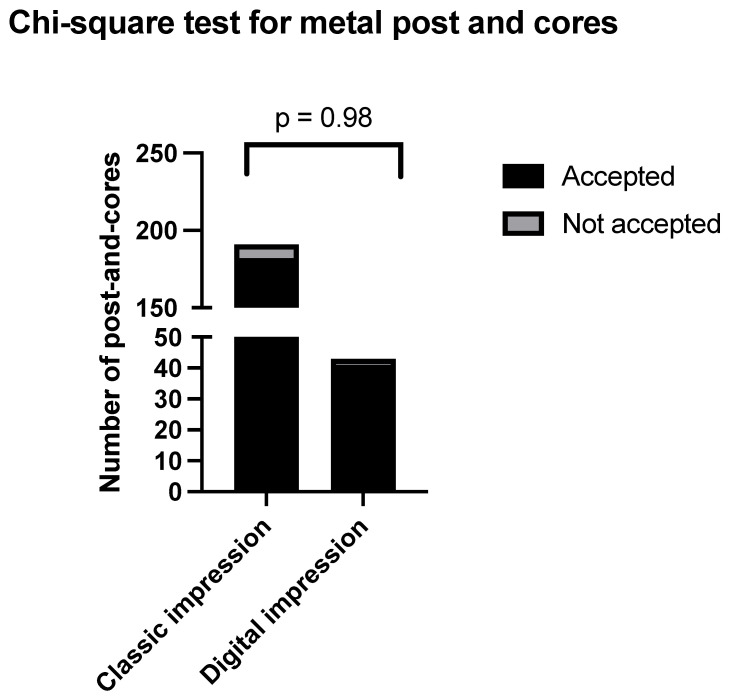
Chi-squared for metal post and cores.

A total of 577 posts were crafted in a dental laboratory over the course of a calendar year. Among them, 234 were metal posts manufactured from Co-Cr, and 343 were zirconia posts. Within the metal category, 191 were created through conventional impression, and 43 were produced through digital impression. Among those produced through conventional impression (Group 1), 182 were accepted by the clinician, and 9 were not accepted. Among those produced through digital im-pression (Group 2), 41 were accepted by the clinician, and 2 were not accepted. Calculating the percentage of clinical acceptability, it can be observed that 95% of metal post and cores obtained from conventional impressions were accepted by the clinician for cementation. A similar percentage, also 95%, was recorded for metal post and cores obtained through digital impressions, signifying clinician approval for cementation. The Chi-squared test yielded a result of *p* = 0.98, indicating no statistically significant difference. The data can be observed in Figure 1.

Within the zirconia category, 224 were created through conventional impression, and 119 were produced through digital impression. Among those produced through conventional impression (Group 3), 213 were accepted by the clinician, and 11 were not accepted. Among those produced through digital impression (Group 4), 105 were accepted by the clinician, and 14 were not accepted.

**Figure 2:**
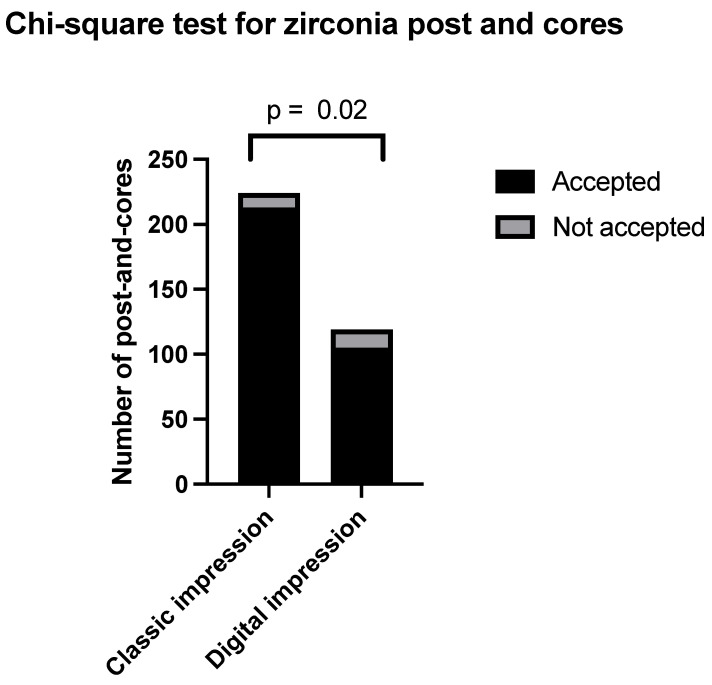
Chi-squared for zirconia post and cores.

The percentage of clinically acceptable zirconia post and cores, calculated from conventional impressions, was observed to be 95%, as accepted by the clinician for cementation. In contrast, a clinical acceptance rate of 88% was recorded for zirconia post and cores obtained through digital impressions. The Chi-squared test resulted in a p-value of 0.02, meaning a statistically significant difference. The relevant data are presented in Figure 2.

Analyzing these data, it can be observed that the null hypothesis formulated at the beginning of this study was rejected only in the case of zirconia posts, where a significant difference in clinical acceptance was noted between those manufactured using conventional impressions versus digital impressions. Thus, a significantly higher acceptance rate is evident among zirconia posts manufactured through conventional impressions. For metal posts, the null hypothesis could not be rejected.

## 4. Discussion

It can be observed that all the articles published so far regarding “clinical acceptance” are about crowns and not about posts. A systematic review conducted by Goujat et al. [17] concluded that most of the studies reported a clinically acceptable range for marginal adaptation (<120 mm). Ferrairo et al. [18] demonstrated that the four tested CAD/CAM systems generated monolithic lithium disilicate restorations with clinically acceptable marginal adaptation and internal fit. Another study on clinically acceptance was conducted by Farah RI et al. [19] who indicated that three investigated CAD programs have the capability to design crowns with clinically acceptable internal and marginal fit.

There is currently no consensus regarding the clinically acceptable value for marginal discrepancy. Some authors propose it to be below 100 μm [20,21,22,23], while others consider a gap less than 120 μm as an appropriate threshold [24,25,26,27,28]. Da Costa et al. [20] concluded that achieving a gap width below 100 μm is desirable and considered clinically acceptable. Norah et al. [21] demonstrated that different fabrication techniques influenced the marginal and internal adaptation of ceramic inlay restorations. The heat-press group exhibited the best marginal and internal adaptation results; however, in every group, all samples were within the clinically acceptable marginal gap limit (100 μm). Roperto et al. [23] also found that differences in milling unit generation did not significantly affect the marginal fit. Marginal gap means fell within the range of clinical acceptance levels for both generations of Cerec milling units, regardless of the teeth site area. Most studies have reported marginal gap values falling within this range (<120 μm). For internal adaptation, proposed values range from 70 μm to 120 μm. Additionally, studies suggest that an internal gap of 50 μm to 100 μm may lead to the most favorable performance of resin cement [29,30,31].

While the use of intraoral scanners for digital scanning of post cavities is acknowledged for its potential to accelerate manufacturing processes, it has been noted that there are still limitations in depth reading with this digital technique. An alternative approach involves the utilization of conventional root canal impressions combined with digital scanning of the dental model obtained by pouring gypsum into the impression, using an extraoral lab scanner, forming a hybrid impression technique [32]. In this study, the hybrid impression technique was utilized, considered an optimal choice for digital scanning.

One of the advantages of custom-made post-and-core restorations lies in their superior adaptation to the unique shape of the root canal preparation. The choice of a suitable material for post-and-core restoration relies on clinical evaluation by a dental specialist, as there is no universally applicable material for all clinical situations. The prevalent use of metal alloys in custom post-and-cores is attributed to their cost-effectiveness and the established protocol for conventional fabrication through casting. CAD/CAM technologies introduce novel methods for processing dental alloys, ensuring proven properties and yielding predictable results. Advancements in digital technologies have led to the development of innovative materials that offer improved opportunities to meet the requirements of both patients and clinicians. The rigidity, modulus of elasticity, and debonding rate of metal alloys and fiber-reinforced composites have been subjects of discussion among researchers, with conflicting results. In a meta-analysis from 2019, Wang et al. [33] studied the difference between fiber posts and metal post and cores in terms of survival, success, post debonding, and root fracture. After assessing 1511 records, 14 full text articles were selected and only four randomized controlled trials met the inclusion criteria. The study concluded that fiber posts presented higher survival rates in comparison with metal post and cores, but no significant difference was observed in terms of success rate—debonding of root fracture rates. However, Sarkis-Onofre et al. [34] concluded that glass-fiber and cast metal posts exhibit similar clinical performance. Moreover, clinicians opt for zirconia post-and-cores in the esthetic zone, aiming to prevent the metal color from showing through ceramic crowns. Although more costly than their metal counterparts, their mechanical properties differ. Therefore, they are not favored for the posterior zone where substantial pressures are exerted.

For both zirconia and metallic post-and-cores, the manufacturing process starts with the impression, which can be recorded either digitally or physically. If the impression will be conventional, an addition silicone material will be used (Elite HD, Zhermack, Badia Polesine, Italy). In certain situations, the impression of the antagonists and occlusion is not necessary. But, if the clinician wants to design the post and core in accordance with the shape of the crown above, antagonists and occlusion must be recorded.

When it comes to the limitations of the addition silicone, impression errors are a big concern because they have the potential to drastically affect how well the final post-and-core fit, which in turn can affect how well therapy goes. It is hypothesized that dentists’ inexperience with the material is what causes the inaccurate impressions. In an attempt to decrease contact angle and increase wettability, external and/or internal surfactants have been added to VPS impression materials to increase their hydrophilicity. Therefore, it should be underlined that reliable impressions can only be obtained in dry conditions.

Pouring the plaster models can result in errors that can affect the final precision of the post and cores. As an alternative to this, the conventional impression can be scanned using a desktop scanner. Subsequently, by using the CAD software, this scan can be digitally inverted so that negative parts become positive. Hence, with a single click, a digital model can be obtained from the scan of the conventional impression.

One of the limitations of this study is the size of the studied sample. For further information, additional studies can be conducted in the future to assess the causes of the lack of fit of posts, similar to crowns.

## 5. Conclusions

A significantly higher acceptance rate is apparent among zirconia post and cores manufactured through conventional impressions, in contrast to zirconia post and cores produced via digital impressions. No statistically significant difference was identified between metal post and cores obtained through digital impressions and those acquired through conventional impressions.

## Data Availability

More data can be found at https://umfcd.ro/educatie/doctorat/scoala-doctorala/ (accessed on 10 June 2024).
